# Association of size at birth with adolescent hormone levels, body size and age at menarche: relevance for breast cancer risk

**DOI:** 10.1038/sj.bjc.6604449

**Published:** 2008-07-01

**Authors:** S Opdahl, T I L Nilsen, P R Romundstad, E Vanky, S M Carlsen, L J Vatten

**Affiliations:** 1 Department of Public Health, Faculty of Medicine, Norwegian University of Science and Technology, Trondheim NO-7489, Norway; 2 Østfold Hospital Trust (Sykehuset Østfold HF), Fredrikstad NO-1603, Norway; 3 Human Movement Science Programme, Faculty of Social Sciences and Technology Management, Norwegian University of Science and Technology, Trondheim NO-7491, Norway; 4 Department of Laboratory Medicine, Children's and Women's Health, Faculty of Medicine, Norwegian University of Science and Technology, Trondheim NO-7491, Norway; 5 Department of Obstetrics and Gynecology, Trondheim University Hospital (St Olav's Hospital), Olav Kyrres gate 17, Trondheim NO-7006, Norway; 6 Department of Endocrinology, Trondheim University Hospital (St Olav's Hospital), Olav Kyrres gate 17, Trondheim NO-7006, Norway

**Keywords:** size at birth, age at menarche, anthropometry, sex steroids, breast cancer risk

## Abstract

Birth size has been positively associated with age at menarche and height in adolescence and adulthood, but the relevant biological mechanisms remain unclear. Among 262 Norwegian term-born singleton girls, birth size measures (weight, length, ponderal index, head circumference and subscapular skin-fold thickness) were analysed in relation to adolescent hormone levels (oestradiol, prolactin, dehydroepiandrosterone sulphate, androstenedione and free testosterone index), age at menarche and adolescent (ages 12.7–15.5 years) and body size (height, weight, body mass index and waist-to-hip ratio) using survival analysis and general linear modelling. The results were adjusted for gestational age at birth, age and menarcheal status at measurement in adolescence and maternal age at menarche. Birth weight, birth length and head circumference were positively associated with adolescent weight and height, and small birth size was associated with earlier age at menarche. Subscapular skin-fold thickness at birth was not associated with adolescent body size, but low fold-thickness was associated with earlier age at menarche. Measures of birth size were inversely related to circulating levels of dehydroepiandrosterone sulphate in adolescence, but there was no clear association with other hormones. These results suggest that physical and sexual development in puberty and adolescence is influenced by prenatal factors, and in combination, these factors may influence health and disease later in life.

The hypothesis that breast cancer (BC) may originate *in utero* (Trichopoulos, 1990) has stimulated studies of birth size in relation to risk factors for BC (Okasha *et al*, 2003). Birth size is an indicator of foetal growth and may reflect intrauterine exposure to oestrogen, the key factor that was originally proposed to explain a possible association between intrauterine factors and adult BC (Trichopoulos, 1990). Studies have shown that relatively large birth size is associated with later age at menarche (Adair, 2001; Sloboda *et al*, 2007) and taller body height, both in adolescence (Pietiläinen *et al*, 2001; Romundstad *et al*, 2003) and in adulthood (Loos *et al*, 2002). Adult tallness is associated with higher BC risk, and the positive association between birth size and body height suggests that longitudinal growth in childhood and adolescence may be important determinants for subsequent BC (Michels and Willett, 2004). Birth size has also been inversely related to adrenal androgen production during childhood (Ong *et al*, 2004) and adolescence (Ibanez *et al*, 1999). Adult levels of both adrenal and gonadal androgens have been associated with BC risk (Kaaks *et al*, 2005a, 2005b). The fact that small birth size is associated with earlier age at menarche and higher circulating levels of adrenal androgens cannot be easily reconciled with known BC risk factors, as small birth size may be related to reduced risk, and both early age at menarche and androgens are expected to increase risk.

We have examined the relation between birth size characteristics and potential BC risk factors (ESHRE Capri Workshop Group, 2004), and specifically the association with age at menarche and height and weight in adolescence, as well as with circulating levels of prolactin, androgens and oestradiol.

## Materials and methods

The study is based on the Norwegian part of an international study among pregnant women in 1985–1986, described in detail elsewhere (Bakketeig *et al*, 1993). Briefly, Caucasian women with one or two singleton births were included. Of these, two groups were followed in detail throughout pregnancy and their children extensively examined at birth, one of a 10% random sample (*n*=561) of all women; the other group of women with one or more of the following risk factors for giving birth to a small birth size for gestational age child (SGA, i.e., in the 10% lowest birth weights for known gestational age): a previous low-birth-weight child or perinatal death, cigarette smoking at conception, pre-pregnancy weight below 50 kg, chronic renal disorder or hypertension (*n*=1384).

In 2001–2002, girls born in the original study were invited to a follow-up examination in adolescence. Among a total of 523 invited girls, 146 were excluded because they were not born at term (before week 37 or after week 42) or because exact gestational age was missing. In addition, 39 girls were excluded either because they had died (*n*=5), were reluctant to participate (*n*=8) or gave practical reasons (*n*=26). Of the remaining 338 invited girls, 262 attended the follow-up examination (77.6%). Mean age at examination in adolescence was 14.0 years (range 12.7–15.5 years). The examination included anthropometric measurements, serum samples and a questionnaire related to health and life style factors, pubertal development and menstrual status. The study was approved by the Regional Committee for Medical Research Ethics and by the Norwegian Data Inspectorate.

Birth size measurements included length (crown to heel, to the nearest half, cm), weight (in g), ponderal index (weight/length^3^, in g cm^−3^), head circumference (in mm) and subscapular skin-fold thickness (10th of a millimetre after 60 s using a Harpenden calliper, placed below the inferior angle of the left scapula). Each measure was categorised into three groups of similar size (tertiles). Information on maternal age at menarche (years) and gestational age at delivery (days) was collected at birth.

Birth size measurements were analysed in relation to the measures of adolescent body size (height, weight, body mass index (BMI) and waist-to-hip ratio) age at menarche and to adolescent levels of oestradiol, prolactin, dehydroepiandrosterone sulphate (DHEAS), androstenedione and also free testosterone index (FTI), calculated as (total testosterone/SHBG) × 100. Analyses of DHEAS and prolactin were carried out in all girls, regardless of menstrual status, but for oestradiol, androstenedione and FTI were restricted to those in the follicular phase of their menstrual cycle at examination (i.e., if the first day of her last menstruation was no more than 12 days before). We excluded six girls who had blood levels of LH higher than 10.0 U l^−1^ and/or 17-OH-progesterone higher than 10.0 nmol l^−1^, and two girls with abnormally high levels of progesterone, leaving 62 girls for these hormone-related analyses.

Testosterone and androstenedione levels were measured by a double antibody technique on an Elecsys 2010 analyser (Roche Diagnostics GmbH, Mannheim, Germany). Levels of oestradiol, progesterone and DHEAS were measured using a competitive immunoassay on an Immulite 2000 analyser (Diagnostic Products Corporation, Los Angeles, CA, USA). Levels of SHBG, LH and prolactin were measured by an immunometric assay on an Immulite 2000 analyser. Reagents and calibrators supplied by the manufacturers were used. 17-OH-Progesterone levels were measured using radioimmunoassay technique with reagents and calibrators supplied by Orion Diagnostica (Espoo, Finland). The lower and upper reference values for women were 0.9 and 11.7 *μ*mol l^−1^ for DHEAS, 0.7 and 11.0 nmol l^−1^ for androstenedione, 0.1 and 2.9 nmol l^−1^ for testosterone and 1.5 and 12.8 nmol l^−1^ for 17-OH-progesterone. The lower detection limits were 0.6 nmol l^−1^ for progesterone, 0.8 *μ*mol l^−1^ for DHEAS and 0.1 nmol l^−1^ for testosterone. The lower and upper detection limits were 0.07 and 7.34 nmol l^−1^ for oestradiol, 3 and 180 nmol l^−1^ for SHBG, 0.05 and 200.00 mIU l^−1^ for LH and 11 and 3180 mIU l^−1^ for prolactin. For each hormone, analyses were performed in a single kit on the same day. For girls with levels of progesterone, oestradiol or DHEAS below the detection limit, levels were set to 0.1 nmol l^−1^, 0.01 nmol l^−1^ and 0.1 *μ*mol l^−1^, respectively.

### Statistical analysis

Median age at menarche for each birth size group was estimated by Kaplan–Meier survival analysis and multivariable analysis was performed using Cox regression. Differences in adolescent body size and hormone levels between groups according to birth size, and linear trends across birth size categories, were evaluated using general linear modelling. Mean values with 95% confidence intervals (CI) within each birth size category are reported. In the hormone analyses, we used logarithmic transformation, as these variables were not normally distributed, and geometric means are therefore reported.

We evaluated potential confounding by BMI and age in adolescence, maternal age at menarche and residential area (Trondheim or Bergen). All analyses of body size and hormone levels in adolescence were adjusted for residential region and age in adolescence. In addition, we adjusted for menarcheal status (pre or post) in the analyses of adolescent body size and levels of prolactin and DHEAS. In other hormone analyses, we adjusted for time as menarche and day of menstrual cycle. All analyses were adjusted for gestational age. To minimise confounding by the relatively high prevalence of risk factors for SGA births, we compared the high-risk group (*n*=195) and the random sample (*n*=67) for all variables included using general linear modelling. We also conducted stratified analyses of these groups for birth size related to age at menarche, adolescent body size and levels of prolactin and DHEAS. All statistical analyses were conducted using the statistical software SPSS for Windows, Release 13.0, Copyright^©^ SPSS Inc., 1989–2004 (Chicago, IL, USA).

## Results

At examination during adolescence (mean age 14.0 years), 199 out of 262 girls (76.0%) had reached menarche (Table 1). For all birth size variables, except ponderal index, girls who were relatively small at birth were younger at menarche than those with larger birth size (Table 2). Median age at menarche for girls in the shortest birth length category (<49.0 cm) was 12.5 years (95% CI, 12.26–12.74), compared with 13.3 years (95% CI, 12.95–13.72) for the highest category (⩾51.0 cm). Similar results were observed for birth weight, whereas the differences in age at menarche for the other birth size variables were smaller. Adjustment for potentially confounding factors did not substantially alter these results (data not shown).

We found that birth length, weight and head circumference were positively associated with adolescent height and weight (Table 2). The associations were further strengthened after adjustment for age at examination in adolescence, menarcheal status and geographical region. Comparing the longest and shortest categories at birth (⩾51 *vs* ⩽49 cm), the longest were on average 6.2 cm taller (164.6 *vs* 158.4 cm) and 4.2 kg heavier (55.0 *vs* 50.8 kg) in adolescence. Girls in the heaviest birth category (⩾3700 g) were 4.4 cm taller and 4.7 kg heavier in adolescence than those with birth weight below 3200 g. For ponderal index, there was a negative association with adolescent height; girls who were thin at birth were on average taller in adolescence, but the association was attenuated after adjustment for age, menarcheal status and residential region. Girls in the highest category of birth weight and head circumference had higher BMI in adolescence compared with girls of average birth size (*P*<0.02, results not shown). None of the birth size variables were related to waist/hip ratio in adolescence, and skin-fold thickness at birth showed no association with any of the anthropometric variables in adolescence (data not shown). Adjustment for gestational age had no influence on the observed associations (results not shown).

A negative association between head circumference at birth and circulating level of prolactin in adolescence (*P*-trend=0.03) was attenuated after adjustment for age at puberty, menarcheal status and residential region (*P*-trend=0.12) (Table 3). For other birth size factors, we found no relation with prolactin. There was a significant negative association between birth weight, head circumference and subscapular skin-fold thickness and circulating levels of DHEAS in adolescence (i.e. lower DHEAS levels with increasing size), unaffected by adjustment for the above factors. Mean (geometric) DHEAS for adolescent girls in the highest birth weight category was 1.95 *μ*mol l^−1^ (95% CI, 1.64–2.32) compared with 2.28 *μ*mol l^−1^ (95% CI, 1.88–2.78) for the lowest category of birth weight.

For girls who were in the follicular phase of the menstrual cycle (Table 4), we found no evidence for any association between birth size and FTI or level of androstenedione in adolescence. For oestradiol, there was a tendency for a weak positive association with all factors of birth size, but significant only for subscapular skin-fold thickness. Adjustment for age at examination, time since menarche and region did not substantially influence these estimates, whereas adjustment for day of menstrual cycle reinforced them.

Comparing girls from the random sample with those whose mothers had higher risk of delivering an SGA child, we found no difference for any of the variables (all *P*-values >0.1, results not shown). Stratified analyses of these groups showed similar results as the overall analyses.

## Discussion

In this study of 262 girls who were followed from birth to adolescence, those whose birth weight, length and head circumference were relatively high had later age at menarche, higher weight and they were taller in adolescence than those with relatively small birth size. In relation to circulating hormone levels in adolescence, greater birth size was associated with relatively lower DHEAS levels; adolescent levels of oestradiol were weakly associated with birth size. For other hormones, we found no clear association with birth size.

Strengths of the present study include the various measures of anthropometry at birth and in adolescence, all standardized and conducted by trained personnel, and the opportunity to adjust for potential confounders such as maternal age at menarche and cyclic variation of hormone levels. Girls who had reached menarche were examined shortly after their first menstrual period, reducing possible recall bias. The modest number of participants is a limitation of the study. For hormone analyses among girls who were in the follicular phase of the menstrual cycle, this reduced the precision of the results.

Our results support the previously reported positive associations of birth size (either birth weight or length), with age at menarche (Adair, 2001; Sloboda *et al*, 2007) and adolescent weight and height (Pietiläinen *et al*, 2001; Romundstad *et al*, 2003), and indicate that the associations may also extend to additional measures of birth size such as head circumference and skin-fold thickness.

Previous studies indicate that SGA children have higher levels of DHEAS before adrenarche (Dahlgren *et al*, 1998), during adolescence (Ibanez *et al*, 1999) and in early adulthood (Szathmari *et al*, 2001). An inverse relation of birth size with adrenal androgen levels in 8-year-old children has also been found for children of normal birth size (Ong *et al*, 2004). In a recent study of hormone levels in premenopausal women, there was a suggestive inverse association of birth weight with DHEAS (Tworoger *et al*, 2006), with which our adolescent DHEAS findings accord. Furthermore, it strengthens the evidence that it is valid for all birth size, and not only for SGA children.

Circulating levels of DHEAS increase during foetal life, but decline rapidly after birth and remain low during early childhood, gradually increasing from about the age of 6 years (adrenarche) through puberty and reaching a peak in early adult life, followed by a continuous decline with age (Auchus and Rainey, 2004). Dehydroepiandrosterone sulphate can be converted both to more potent androgens and to oestrogens (Ibanez *et al*, 2000; Nicolas Diaz-Chico *et al*, 2007), but its physiological role is unclear and has been hypothesised to depend on the hormonal milieu (Ebeling and Koivisto, 1994). The physiological triggers of adrenarche and adrenal androgen production are unknown, although there are indications that ACTH, insulin, IGF-I and leptin may be implicated (Ibanez *et al*, 2000).

Our findings could be interpreted both as further indications of the earlier maturation previously reported in children of small birth size and as a potentially lifelong influence of developmental plasticity on adrenal androgen production. The increase in concentration of DHEAS parallels the increase in skeletal age through puberty (Ibanez *et al*, 2000). Thus, adrenal androgen production may contribute to the relation between birth size and adolescent height, as small birth size is associated with both higher DHEAS levels and a lower adolescent and final height.

Oestrogens are believed to be involved in BC development, both prenatally (Trichopoulos, 1990) and later in life (ESHRE Capri Workshop Group, 2004). We observed a weak tendency for a positive association between birth size and adolescent oestradiol level. In two previous studies, both of which controlled for cyclic variation, positive associations of premenopausal oestradiol levels with ponderal index at birth (Jasienska *et al*, 2006) and birth weight (Tworoger *et al*, 2006) were reported. If this relation is true, it may be in accord with the positive association of birth size with BC risk observed in many studies.

Longitudinal growth in childhood may be positively associated with subsequent BC risk (De Stavola *et al*, 2004), and the association may be independent of birth weight (Ahlgren *et al*, 2004; dos Santos Silva *et al*, 2004). High childhood growth rate is also associated with earlier age at menarche (Sloboda *et al*, 2007), and childhood growth patterns influence the association between birth size and age at menarche (Adair, 2001; dos Santos Silva *et al*, 2002). A large population-based study from Denmark (Ahlgren *et al*, 2004) found no effect of age at menarche on BC risk after adjustment for childhood growth patterns.

There are only weak indications that the inverse relation of birth size with adrenal androgen production in childhood and adolescence continues into adulthood (Szathmari *et al*, 2001; Tworoger *et al*, 2006). However, should it do so, this would be in conflict with the association between BC risk and the levels of adrenal androgens in adulthood (Kaaks *et al*, 2005a, 2005b), as a smaller birth size is associated with reduced risk. As for age at menarche, opposing influences of birth size and childhood growth, weight gain and overweight have also been reported for adrenal androgen production in children (Ong *et al*, 2004), indicating that the combined pattern of prenatal and postnatal growth may define the subsequent risk profile.

In conclusion, relatively low birth size was associated with earlier age at menarche and larger body size in adolescence, but negatively related to adolescent DHEAS level. The results demonstrate a link between prenatal factors and subsequent growth and development.

## Figures and Tables

**Table 1:** Mean values (s.d.), minimum and maximum values and number of missing values of selected variables among 262 Norwegian girls

**Variables**	**Mean (s.d.)**	**Range**	**Missing (*n*)**
*At birth*			
Birth weight (g)	3463 (476)	2420–4710	0
Birth length (cm)	50.0 (1.8)	42.0–55.8	3
Ponderal index (g cm^−3^)	2.76 (0.26)	2.09–4.18	3
Head circumference (cm)	34.8 (1.2)	31.9–38.0	2
Skin-fold thickness (mm)	4.4 (1.0)	2.2–7.9	3
Gestational age (days)	282.6 (7.1)	264.0–294.0	0
			
*In adolescence*			
Age (years)	14.0 (0.7)	12.7–15.5	0
Weight (kg)	54.5 (9.9)	33.9–96.4	2
Height (cm)	162.9 (6.8)	143.0–183.5	1
BMI (kg/m^2^)	20.5 (3.0)	14.3–32.0	2
Waist/hip ratio	0.78 (0.06)	0.63–1.26	1
Age at menarche (years)^a^	13.1 (0.1)	9.5–14.5	6
			
*Maternal*			
Age at menarche (years)	13.0 (1.4)	10.0–17.0	0

a Median age at menarche (standard error), calculated by the use of Kaplan–Meier survival analysis.

**Table 2:** Adolescent anthropometric measures and age at menarche by birth size among 262 Norwegian girls

		**Mean^a^ (95% CI)**		**Median (95% CI)**
**Variables**	**No. of girls**	**Height (cm)**	**Weight (kg)**	**Age at menarche (years)**
*Birth length (cm)*				
<49.0	84	158.4 (157.0–159.7)	50.8 (48.6–53.0)	12.50 (12.26–12.74)
49.0–51.0	84	160.8 (159.5–162.1)	51.7 (49.6–53.8)	13.08 (12.94–13.22)
⩾51.0	91	164.6 (163.4–165.8)	55.0 (53.1–56.9)	13.33 (12.95–13.72)
Trend test^b^		*β*=3.13, *P*<0.001	*β*=2.12, *P*=0.003	*P*<0.001
				
*Birth weight (g)*				
<3200	80	159.9 (158.5–161.4)	51.8 (49.6–53.9)	12.58 (12.32–12.84)
3200–3700	101	160.4 (159.2–161.7)	50.0 (48.1–51.9)	13.25 (12.94–13.56)
⩾3700	81	164.3 (162.9–165.6)	56.5 (54.5–58.5)	13.33 (12.97–13.70)
Trend test^b^		*β*=2.16, *P*<0.001	*β*=2.37, *P*=0.001	*P*=0.001
				
*Ponderal index (g cm*^−*3*^)				
<2.63	83	162.4 (160.9–163.8)	52.7 (50.5–54.8)	12.83 (12.53–13.14)
2.63–2.85	93	161.8 (160.4–163.2)	52.0 (50.0–54.1)	13.08 (12.82–13.35)
⩾2.85	83	160.8 (159.3–162.2)	53.6 (51.5–55.8)	13.17 (12.68–13.65)
Trend test^b^		*β*=−0.81, *P*=0.098	*β*=0.47, *P*=0.52	*P*=0.099
				
*Head circumference (cm)*				
<34.2	86	160.5 (159.0–161.9)	51.9 (49.8–54.0)	12.83 (12.42–13.25)
34.2–35.4	92	160.8 (159.5–162.1)	51.0 (49.0–52.9)	13.42 (13.03–13.81)
⩾35.4	82	163.4 (162.0–164.8)	55.2 (53.2–57.3)	13.17 (12.62–13.71)
Trend test^b^		*β*=1.45, *P*=0.003	*β*=1.66, *P*=0.020	*P*=0.003
				
*Skin-fold thickness (mm)*				
<3.8	88	161.5 (160.0–162.9)	52.2 (50.1–54.4)	12.83 (12.53–13.13)
3.8–4.8	88	161.3 (159.9–162.7)	52.5 (50.4–54.5)	12.92 (12.46–13.37)
⩾4.8	83	161.9 (160.4–163.3)	53.2 (51.1–55.3)	13.50 (13.07–13.93)
Trend test^b^		*β*=0.18, *P*=0.72	*β*=0.50, *P*=0.49	*P*=0.001

CI=confidence interval.

a Adjusted for age at puberty, menarche status and region.

b Regression coefficient, *β*, denoting increase in adolescent size measure with increasing birth size category and corresponding *P*-value.

**Table 3:** Adolescent hormone levels by birth size among 262 Norwegian girls

		**Geometric mean^a^ (95 % CI)**	
**Variables**	**No. of girls**	**Prolactin (mIU l^−1^)**	**DHEAS (*μ*mol l^−1^)**
*Birth length (cm)*			
<49.0	84	179.9 (162.1–199.6)	2.28 (1.88–2.78)
49.0–51.0	84	206.1 (186.9–227.3)	2.09 (1.73–2.51)
⩾51.0	91	176.8 (161.1–193.9)	1.95 (1.64–2.32)
Trend test^b^		*e^β^*=0.99, *P*=0.74	*e^β^*=0.92, *P*=0.21
			
*Birth weight (g)*			
<3200	80	184.6 (165.7–205.6)	2.51 (2.06–3.05)
3200–3700	101	188.7 (171.8–207.3)	2.08 (1.75–2.46)
⩾3700	81	186.6 (168.6–206.5)	1.83 (1.52–2.20)
Trend test^b^		*e^β^*=1.01, *P*=0.88	*e^β^*=0.85, *P*=0.015
			
*Ponderal index (g cm*^−*3*^)			
<2.63	83	180.5 (163.3–199.6)	2.25 (1.86–2.71)
2.63–2.85	93	197.6 (179.8–217.2)	2.16 (1.81–2.58)
⩾2.85	83	181.7 (164.1–201.2)	1.84 (1.52–2.23)
Trend test^b^		*e^β^*=1.00, *P*=0.90	*e^β^*=0.91, *P*=0.13
			
*Head circumference (cm)*			
<34.2	86	195.5 (176.2–216.9)	2.47 (2.03–2.99)
34.2–35.4	92	188.4 (171.5–207.1)	2.03 (1.70–2.42)
⩾35.4	82	175.7 (159.3–193.9)	1.92 (1.60–2.30)
Trend test^b^		*e^β^*=0.95, *P*=0.12	*e^β^*=0.88, *P*=0.047
			
*Skin-fold thickness (mm)*			
<3.8	88	194.7 (175.6–215.9)	2.41 (2.00–2.91)
3.8–4.8	88	181.8 (164.7–200.6)	2.19 (1.83–2.62)
⩾4.8	83	185.5 (167.2–205.8)	1.73 (1.43–2.09)
Trend test^b^		*e^β^*=0.98, *P*=0.48	*e^β^*=0.85, *P*=0.012

CI=confidence interval; DHEAS=dehydroepiandrosterone sulphate.

a Adjusted for age at puberty, menarche status and region.

b *e^β^* × 100% equals percent change in hormone levels with increasing birth size category and corresponding *P*-value.

**Table 4:** Adolescent hormone levels by birth size among 62 Norwegian girls in the follicular phase^a^ of the menstrual cycle

		**Geometric mean^b^ (95 % CI)**		
**Variables**	**No. of girls**	**Free testosterone index**	**Androstenedione (nmol l^−1^)**	**Estradiol (nmol l^−1^)**
*Birth length (cm)*				
<49.0	26	2.16 (1.61–2.90)	8.0 (7.1–9.1)	0.08 (0.05–0.12)
49.0–51.0	17	2.17 (1.50–3.13)	8.2 (7.1–9.6)	0.10 (0.06–0.17)
⩾51.0	19	2.15 (1.53–3.02)	8.2 (7.1–9.4)	0.12 (0.07–0.19)
Trend test^c^		*e^β^*=1.00, *P*=0.98	*e^β^*=1.01, *P*=0.81	*e^β^*=1.24, *P*=0.17
				
*Birth weight (g)*				
<3200	26	2.01 (1.52–2.66)	7.9 (7.0–8.9)	0.08 (0.05–0.12)
3200–3700	21	2.64 (1.93–3.61)	8.4 (7.4–9.6)	0.10 (0.06–0.16)
⩾3700	15	1.83 (1.26–2.67)	8.3 (7.1–9.7)	0.13 (0.07–0.21)
Trend test^c^		*e^β^*=0.98, *P*=0.90	*e^β^*=1.03, *P*=0.54	*e^β^*=1.28, *P*=0.14
				
*Ponderal index (g cm*^−*3*^)				
<2.63	24	2.04 (1.53–2.72)	8.2 (7.2–9.2)	0.08 (0.05–0.13)
2.63–2.85	18	2.63 (1.84–3.77)	7.8 (6.7–9.1)	0.10 (0.06–0.17)
⩾2.85	20	1.98 (1.42–2.75)	8.4 (7.3–9.6)	0.11 (0.07–0.18)
Trend test^c^		*e^β^*=0.99, *P*=0.92	*e^β^*=1.01, *P*=0.82	*e^β^*=1.15, *P*=0.39
				
*Head circumference (cm)*				
<34.2	24	2.03 (1.51–2.72)	7.8 (6.9–8.8)	0.09 (0.06–0.15)
34.2–35.4	23	2.34 (1.68–3.24)	8.1 (7.1–9.2)	0.09 (0.06–0.15)
⩾35.4	14	2.12 (1.44–3.12)	8.8 (7.5–10.3)	0.10 (0.06–0.17)
Trend test^c^		*e^β^*=1.03, *P*=0.78	*e^β^*=1.06, *P*=0.23	*e^β^*=1.01, *P*=0.94
				
*Skin-fold thickness (mm)*				
<3.8	25	2.19 (1.64–2.91)	8.2 (7.2–9.2)	0.07 (0.05–0.11)
3.8–4.8	21	1.62 (1.16–2.27)	7.4 (6.4–8.5)	0.11 (0.07–0.17)
⩾4.8	14	2.99 (2.02–4.44)	9.2 (7.7–10.8)	0.14 (0.08–0.25)
Trend test^c^		*e^β^*=1.13, *P*=0.33	*e^β^*=1.05, *P*=0.40	*e^β^*=1.43, *P*=0.049

CI=confidence interval.

a Girls who were measured between day 1 and day 12 of their menstrual cycle and who had LH<10 U l^−1^, 17-OH-progesterone <10 nmol l^−1^ and progesterone <5 nmol l^−1^.

b Adjusted for age at puberty, time since menarche, day of menstrual cycle and district.

c *e^β^* × 100% equals percent change in hormone levels with increasing birth size category and corresponding *P*-value.
